# Supplementary material for: Effects of the COVID-19 pandemic on antenatal care utilisation in Kenya: a cross-sectional study
Source: BMJ Open. 2022 Apr 13;12(4):e060185. doi: 10.1136/bmjopen-2021-060185 (PMC9013791; doi:10.1136/bmjopen-2021-060185)
Supplement: Author's manuscript [file bmjopen-2021-060185.draft_revisions.pdf]

BMJ Open is committed to open peer review. As part of this commitment we make the peer review history of every article we publish publicly available.

When an article is published we post the peer reviewers' comments and the authors' responses online. We also post the versions of the paper that were used during peer review. These are the versions that the peer review comments apply to.

The versions of the paper that follow are the versions that were submitted during the peer review process. They are not the versions of record or the final published versions. They should not be cited or distributed as the published version of this manuscript.

BMJ Open is an open access journal and the full, final, typeset and author-corrected version of record of the manuscript is available on our site with no access controls, subscription charges or pay-per-view fees (http://bmjopen.bmj.com).

If you have any questions on BMJ Open's open peer review process please email info.bmjopen@bmj.com

The Impacts of the COVID-19 Pandemic on Antenatal Care Utilization: A Cross-Sectional Study in Kenya

Journal:	*BMJ Open*
Manuscript ID	bmjopen-2021-060185
Article Type:	Original research
Date Submitted by the Author:	14-Dec-2021

Complete List of Authors:	Landrian, Amanda; University of California Los Angeles<br>Mboya, John; Innovations for Poverty Action Kenya<br>Golub, Ginger; Innovations for Poverty Action Kenya<br>Moucheraud, Corrina; University of California Los Angeles<br>Kepha, Stella; Kenya Medical Research Institute<br>Sudhinaraset, May; University of California Los Angeles

Keywords:	COVID-19, OBSTETRICS, PUBLIC HEALTH

I, the Submitting Author has the right to grant and does grant on behalf of all authors of the Work (as defined in the below author licence), an exclusive licence and/or a non-exclusive licence for contributions from authors who are: i) UK Crown employees; ii) where BMJ has agreed a CC-BY licence shall apply, and/or iii) in accordance with the terms applicable for US Federal Government officers or employees acting as part of their official duties; on a worldwide, perpetual, irrevocable, royalty-free basis to BMJ Publishing Group Ltd ("BMJ") its licensees and where the relevant Journal is co-owned by BMJ to the co-owners of the Journal, to publish the Work in this journal and any other BMJ products and to exploit all rights, as set out in our licence.

The Submitting Author accepts and understands that any supply made under these terms is made by BMJ to the Submitting Author unless you are acting as an employee on behalf of your employer or a postgraduate student of an affiliated institution which is paying any applicable article publishing charge ("APC") for Open Access articles. Where the Submitting Author wishes to make the Work available on an Open Access basis (and intends to pay the relevant APC), the terms of reuse of such Open Access shall be governed by a Creative Commons licence – details of these licences and which Creative Commons licence will apply to this Work are set out in our licence referred to above.

Other than as permitted in any relevant BMJ Author's Self Archiving Policies, I confirm this Work has not been accepted for publication elsewhere, is not being considered for publication elsewhere and does not duplicate material already published. I confirm all authors consent to publication of this Work and authorise the granting of this licence.

For peer review only

The Impacts of the COVID-19 Pandemic on Antenatal Care Utilization: A Cross-Sectional Study in Kenya

Amanda Landrian[1], John Mboya[2], Ginger Golub[2], Corrina Moucheraud[1], Stella Kepha,[3] May Sudhinaraset[1]

[1]Jonathan and Karin Fielding School of Public Health, University of California, Los Angeles, Los Angeles, California, USA

[2]Innovations for Poverty Action, Nairobi, Kenya

[3]Kenya Medical Research Institute, Nairobi, Kenya

*Corresponding Author: Amanda Landrian, Jonathan and Karin Fielding School of Public Health, University of California, Los Angeles, 650 Charles E Young Drive South, Los Angeles, California, United States 90095; alandrian2@gmail.com

Word Count: 3,269

For peer review only - http://bmjopen.bmj.com/site/about/guidelines.xhtml

Abstract

Objective: The aim of this study was to assess the impacts of COVID-19 on antenatal care (ANC) utilization in Kenya, including women's reports of COVID-related barriers to ANC and correlates at the individual and household levels.

Design: Cross-sectional study.

Setting: Six public and private health facilities and associated catchment areas in Nairobi and Kiambu Counties in Kenya

Participants: Data were collected from 1,729 women, including 1,189 women who delivered in healthcare facilities before the COVID-19 pandemic (from September 2019-January 2020) and 540 women who delivered during (from July through November 2020). Women who delivered during COVID-19 were sampled from the same catchment areas as the original sample of women who delivered before to compare ANC utilization.

Primary and secondary outcome measures: Timing of ANC initiation, number of ANC visits, and adequate ANC utilization were primary outcome measures. Among only women who delivered during COVID-19 only, we explored women's reports of the pandemic having affected their ability to access or attend ANC as a secondary outcome of interest.

Results: Women who delivered during COVID had significantly higher odds of delayed ANC initiation (i.e., beginning ANC during the second vs. first trimester) than women who delivered before COVID (aOR 1.72, 95% CI 1.24-2.37), although no differences were detected in the number of visits attended. Nearly half (47%) of women who delivered during COVID-19 reported that the pandemic affected their ability to access ANC.

Conclusions: Strategies are needed to mitigate disruptions to ANC among pregnant women during pandemics and other public health, environmental, or political emergencies.

For peer review only - http://bmjopen.bmj.com/site/about/guidelines.xhtml

Strengths and limitations of this study

- This study provides evidence of COVID-related impacts to antenatal care utilization, a critical determinant of maternal and newborn health, among pregnant women in Kenya.
- This study leveraged existing survey data among postpartum women who delivered just prior to the declaration of the COVID-19 pandemic and recruited a new cohort of women who delivered during the pandemic to explore potential differences in ANC initiation, number of visits, and adequate ANC utilization across the two samples.
- Despite sampling women who delivered during the pandemic from the same catchment areas as those facilities where women who delivered before COVID-19 were sampled, the two samples may not be equivalent and, thus, unmeasured differences in sample characteristics may explain study findings.
- The study design does not allow for a causal interpretation of findings.

For peer review only

For peer review only - http://bmjopen.bmj.com/site/about/guidelines.xhtml

Introduction

Timely and comprehensive antenatal care (ANC) is critical for the health of women and their newborns, allowing for the early detection and management of pre-existing conditions and pregnancy-related complications and reducing the risk of maternal and infant morbidity and mortality (Islam & Tabassum, 2021; Kuhnt & Vollmer, 2017; Pervin et al., 2012; Tekelab et al., 2019; World Health Organization, 2016). The World Health Organization recommends a minimum of eight ANC visits during a woman's pregnancy, with the first visit occurring during the first trimester of gestation (World Health Organization, 2016); however, significant barriers continue to exist for adequate ANC. In Kenya, site of the present study, only 58% of women reported attending at least 4 ANC visits in the most recent Demographic and Health Survey conducted in 2014 (Kenya National Bureau of Statistics et al., 2015).

The COVID-19 pandemic has been extremely disruptive to health systems and services worldwide. Early data indicate that the pandemic has decreased women's use of ANC (Townsend et al., 2021), including in low- and middle-income countries (LMICs) (Burt et al., 2021; Goyal et al., 2021; Kassie et al., 2021). The COVID-19 pandemic has also worsened maternal and perinatal outcomes, particularly for vulnerable groups in LMICs (Chmielewska et al., 2021), but more information is needed about changes in care-seeking patterns during this period. Additionally, much of the available data have focused on overall volume of ANC services without differentiating between timing of initiation and total number of visits or examining heterogeneity in changes to better understand who was most affected by these pandemic-related disruptions. It is also important to understand the enduring effects of how COVID-19 may affect care-seeking. For example, even one year after the 2014-15 Ebola outbreak in West Africa, use of ANC had not yet returned to pre-outbreak levels (Delamou et al., 2017).

Previous research has highlighted the range of mechanisms through which a pandemic might affect health care-seeking behavior (Yerger et al., 2020), including individual level factors such as reduced ability to pay for care if household income is affected by the pandemic, facility-level factors such as closures, health worker shortages, or entry requirements (use of masks and testing), and policy-level factors like restrictions on movement. Understanding how these complex factors may affect women's decisions around ANC is critical to developing appropriate interventions for encouraging care-seeking. Outside the context of a pandemic, Kenyan women from less-wealthy households, lower levels of educational attainment, and those of younger ages may be less likely to achieve adequate ANC (Magadi et al., 2000; Ronen et al., 2017; Wairoto et al., 2020). It is important to understand how these social determinants of health, and other underlying risk factors, may intersect with the COVID-19 pandemic to affect antenatal care-seeking.

Using survey data among women who delivered before and during the COVID pandemic, the primary objective of this paper is to assess the impacts of COVID-19 on the utilization of ANC by examining whether there were reported changes in ANC use before versus during the pandemic. This paper also describes women's reports of the specific ways COVID-19 affected

For peer review only - http://bmjopen.bmj.com/site/about/guidelines.xhtml

their ability to attend ANC and the individual, household, and facility-level factors associated with women's likelihood of reporting COVID-19 to have impacted ANC access or utilization.

Methods

Study participants and recruitment

This study uses cross-sectional data from two samples of participants: 1) Women recruited within seven days of delivery from one of six participating facilities (3 public hospitals, 2 private hospitals, and 1 health center) in Nairobi and Kiambu Counties from September 2019 through January 2020 (i.e., prior to the onset of the COVID-19 pandemic; n=1,197) (Sudhinaraset et al., 2021) and 2) Women residing in catchment areas of these same six participating facilities, who delivered since pandemic-related restrictions were mandated in Kenya (i.e., from March 16, 2020; N=1,135). In both samples, eligible participants were those aged 15-49 years who had delivered a singleton birth within the specified timeframe and had access to a functional phone to allow for follow-up. Additional eligibility criteria for the sample of women who delivered before COVID-19 can be found elsewhere (Sudhinaraset et al., 2021). The sample of women who delivered during COVID-19 was recruited through engagement with community health volunteers and local village leaders and completed the survey in November 2020; among the 1,182 women contacted by phone, a total of 1,135 consented and enrolled in the study (96.0%).

An experienced team of nine female enumerators participated in a three-day, virtual training on the study protocol and survey tools. This was followed by a one-day piloting exercise among 30 women for the enumerators to practice the study consent, assess and refine the survey flow, and test study logistics and quality check procedures. Participants were contacted by phone for both the consent and a one-time, 30-minute survey, though participants had the option for scheduling a separate time for the survey to be administered. For those unable to be reached, a total of 9 attempts were made across different days and times. Participants received the equivalent of approximately $1.00 (United States Dollar) of airtime as a token of appreciation.

Survey measures

The primary outcomes of interest were: timing of ANC initiation, total number of ANC visits, and adequate ANC utilization. The timing of ANC initiation was measured by asking women approximately how many months or weeks pregnant they were when they attended their first ANC appointment. A categorical variable was then created to capture if ANC began in the first, second, or third trimester. The total number of ANC visits was a categorical variable capturing whether women attended <4, 4-7, or ≥8 visits. Finally, information on the timing of ANC initiation and the total number of ANC visits was used to create a binary variable capturing whether women achieved adequate ANC utilization, defined as initiating ANC during the first trimester *and* attending at least 4 visits (1=yes, 0=no).

Among women who delivered during COVID-19 only, we explored whether women reported the pandemic to have affected their ability to access or attend ANC (1=yes, 0=no) as a secondary outcome of interest.

For peer review only - http://bmjopen.bmj.com/site/about/guidelines.xhtml

We also included information on individual and household sociodemographic characteristics, including age, marital status, educational attainment, employment status, self-rated health, and parity. Women who delivered during COVID-19 were asked about household food insecurity using the Household Food Insecurity Access Scale (Coates et al., 2007), and assigned a score (ranging 0-6) reflecting how many household food insecurity indicators they endorsed. Women were also asked how the pandemic affected their ability to access or attend ANC.

Analyses

The analytic sample was first restricted to those with complete information on ANC measures (n=8 missing among women who delivered before COVID-19 and n=13 missing among women who delivered during COVID-19). To ensure that a substantial portion of the gestational period occurred during the pandemic (as opposed to a significant period of gestation occurring prior to the start of the COVID-19 pandemic and/or during the strictest lockdown measures) and would thus be vulnerable to potential COVID-related impacts to ANC utilization, the sample of women who delivered during COVID-19 was further restricted to those who delivered from July 2020 through the end of the study in November 2020. This resulted in an analytic sample of 1,189 women who delivered before and 540 women who delivered during COVID-19.

Data were analyzed using descriptive, bivariate, and multivariable statistics using StataSE version 15. Pearson chi-square tests were used to examine differences in the distribution of demographic characteristics and measures of ANC utilization across study samples. Multivariable logistic regression models were used to assess the relationship between study sample and timing of ANC initiation, number of ANC visits, and adequate ANC utilization, respectively, after controlling for individual level characteristics. Sensitivity analyses were conducted to examine the robustness of the models when restricting the sample of women who delivered during months when lockdown measures and enforcement were most severe (i.e., March through July 2020) to those who delivered from August through November 2020 (N=372) and then September through November 2020 (N=234), respectively.

A multivariable logistic regression model was also used to assess factors associated with women reporting COVID-19 to affect accessing or attending ANC.

Ethical considerations

The Institutional Review Boards at the University of California, Los Angeles (UCLA) and Kenya Medical Research Institute (KEMRI) approved all study procedures and all women provided verbal consent.

Patient and public involvement

Patients and members of the public were not involved in the design of this research; however, members of the public, including community health volunteers and local village leaders in study catchment areas, were involved in the recruitment of women who had delivered during the pandemic. These members of the public were also provided a policy brief of key study findings to disseminate to stakeholders within their communities.

For peer review only - http://bmjopen.bmj.com/site/about/guidelines.xhtml

Results

Descriptive statistics of demographic characteristics stratified by study sample are shown in Table 1. Women who delivered during COVID-19 were older (33% vs. 21% aged at least 30 years; p<0.001), less likely to be married or partnered (69% vs. 83%; p<0.001), more likely to have a secondary education or higher (46% vs. 17%; p<0.001), and less likely to rate their health as excellent, very good, or good (67% vs. 87%; p<0.001) than women who delivered before COVID-19. A significantly lower proportion of women who delivered during the pandemic were employed at the time of the survey than those who delivered before (16% vs. 40%; p<0.001). Compared to women who delivered before COVID-19, those who delivered during were more likely to have 2 or more total births (74% vs. 63%; p<0.001). The mean household food insecurity index score for women who delivered during COVID was nearly 4 (standard deviation=2).

Table 1. Individual and household characteristics of women who delivered before and during the COVID-19 pandemic

Characteristic	**Women who delivered *before* COVID N=1,189**	**Women who delivered *during* COVID N=540**	**p-value[1]**
**Age (years)**			<0.001
Less than 25	576 (48.4)	197 (36.5)	
25-29	364 (30.6)	163 (30.2)	
30-34	170 (14.3)	124 (23.0)	
35 and older	79 (6.6)	56 (10.4)	
**Married or partnered (yes)**	983 (82.7)	374 (69.3)	<0.001
**Educational attainment**			<0.001
Primary or less	526 (44.2)	202 (37.4)	
Some secondary	467 (39.3)	91 (16.9)	
Secondary	165 (13.9)	189 (35.0)	
College/University	31 (2.6)	58 (10.7)	
**Currently employed (yes)**	476 (40.0)	88 (16.3)	<0.001
**Self-rated health status**			<0.001
Fair, poor, or very poor	157 (13.2)	179 (33.2)	
Excellent, very good, or good	1,032 (86.8)	361 (66.9)	
**Parity**			<0.001
1	441 (37.1)	141 (26.1)	
2 or more	748 (62.9)	339 (73.9)	
**Household food insecurity index[2], mean (SD)**	NA	3.7 (1.9)	NA

Note: Frequency (proportion) shown unless otherwise noted. Percentages may not add to 100 due to rounding.
SD = standard deviation. NA = Not applicable.
[1]Pearson chi-squared test
[2]Household food insecurity index denotes the number of household food insecurity indicators endorsed; possible scores range from 0 to 6.

For peer review only - http://bmjopen.bmj.com/site/about/guidelines.xhtml

Table 2 provides descriptive statistics of ANC utilization measures stratified by study sample. Most women in both study samples attended any ANC. A higher proportion of women who delivered before COVID-19 initiated ANC in the first trimester than women who delivered during (21% vs. 15%; p=0.002). No statistically significant differences in the number of ANC visits attended were detected across study samples; most women who delivered before and during COVID-19 attended 4 to 7 visits (61% vs. 60%, respectively). Finally, about 20% of women who delivered before the pandemic achieved adequate ANC utilization compared to 14% of women who delivered during (p=0.002).

Table 2. Utilization of antenatal care (ANC) among women who delivered before and during the COVID-19 pandemic

Characteristic	**Women who delivered *before* COVID N=1,189**	**Women who delivered *during* COVID N=540**	**p-value[1]**
**Attended any ANC, yes**	1,181 (99.3)	534 (98.9)	0.346
**Timing of ANC initiation**			0.002
First trimester	252 (21.2)	81 (15.0)	
Second trimester	777 (65.4)	425 (78.7)	
Third trimester or never	160 (13.5)	34 (6.3)	
**Number of ANC visits**			0.277
Less than 4	439 (36.9)	187 (34.6)	
4-7	717 (60.3)	331 (61.3)	
8 or more	33 (2.8)	22 (4.1)	
**Adequate ANC utilization[2], yes**	238 (20.0)	74 (13.7)	0.002

Note: Frequency (proportion) shown. Percentages may not add to 100 due to rounding.
ANC = antenatal care.
[1]Pearson chi-squared test
[2]Defined as initiating ANC during the first trimester *and* attending at least 4 ANC visits.

Results from logistic regression models assessing the relationship between study sample and measures of ANC utilization are shown in Table 3. After controlling for other individual level characteristics, women who delivered during the pandemic had significantly higher odds of initiating ANC in the second versus first trimester than women who delivered before (adjusted odds ratio [aOR] 1.72, 95% confidence interval [CI] 1.24-2.37). No significant differences in the odds of attending 4-7 or ≥8 ANC visits versus <4 ANC visits, respectively, were detected across the study samples. Women who delivered during COVID-19 had significantly lower odds of achieving adequate ANC utilization than women who delivered before after controlling for individual level characteristics (aOR 0.62, 95% CI 0.44-0.86). Findings did not substantively differ in sensitivity analyses restricting women who delivered during COVID-19 to those whose births occurred from August through November and September through November, respectively.

Table 3. Logistic regression adjusted odds ratios (95% confident intervals) of antenatal care (ANC) outcomes by study sample

For peer review only - http://bmjopen.bmj.com/site/about/guidelines.xhtml

For peer review only

Sample	Timing of ANC Initiation: Second trimester vs. First trimester	Timing of ANC Initiation: Third trimester or never vs. First trimester	Number of ANC Visits: 4-7 vs. Less than 4	Number of ANC Visits: 8 or more vs. Less than 4	Adequate ANC Utilization
Women who delivered *before* COVID-19	Ref	Ref	Ref	Ref	Ref
Women who delivered *during* COVID-19	1.72 (1.24-2.37)**	0.60 (0.36-1.00)	1.12 (0.86-1.44)	1.46 (0.74-2.86)	0.62 (0.44-0.86)**

Note: Timing of ANC initiation and number of ANC visits use multinomial logistic regression, while adequate ANC utilization uses multivariable logistic regression. All models are adjusted for individual characteristics including women's age, marital status, education, employment status, self-rated health status, and parity. ANC = antenatal care.
*p<0.05, **p<0.01

Women who delivered during COVID-19 were asked to report how the pandemic affected their ability to access or attend ANC (Table 4). Nearly half (47%) of all women reported *any* impacts to ANC due to COVID-19. Among these women (N=255), the most reported impacts included facilities being closed, too busy, or not accepting patients (61%), being scared to contract COVID-19 if going to a hospital or health facility (20%) or going out into the community (15%), an inability to afford care because of COVID-19 (15%), and COVID-related restrictions, such as curfews or mask mandates, hindering ANC access (12%).

Table 4. Reported COVID-related impacts to antenatal care utilization among women who delivered during COVID

Impacts	**Women who delivered *during* COVID-19 (N=540)**
**Reported COVID-19 to affect accessing or attending ANC**	
Yes	255 (47.2)
No	285 (52.8)
**Among those who reported COVID-19 to affect accessing or attending ANC (N=255)[1]**	
Facility was closed, too busy, or not accepting patients	156 (61.2)
Scared to get COVID if going to hospital/health facility	50 (19.6)
Could not afford care because of COVID	38 (14.9)
Scared to get COVID if going out into community	37 (14.5)
COVID-related restrictions (e.g., curfew, mask mandate)	30 (11.8)
Scared of police or other officials	8 (3.1)
Inability to pay for or find transportation	7 (2.8)
Do not trust health facility right now	4 (1.6)

Note: Frequency (proportion) shown. ANC = antenatal care.
[1]Responses are not mutually exclusive.

Table 5 provides results of the logistic regression model examining associations between individual and household level characteristics and the odds of women reporting COVID-19 to have affected their ability to access or attend ANC. A significant association was found between

For peer review only - http://bmjopen.bmj.com/site/about/guidelines.xhtml

educational attainment and reporting COVID-related impacts; increasing education was associated with increasing odds of reporting COVID-19 to affect women's ability to access or attend ANC compared to those with a primary education or less. Women who rated their health as excellent, very good, or good had an odds of reporting COVID-related impacts to ANC that was about 50% lower than women who rated their health as fair, poor, or very poor (aOR 0.51, 95% CI 0.34-0.75). Compared to women with only one birth, women with 2 or more births had significantly higher odds of reporting COVID-19 to affect accessing or attending ANC (aOR 1.84, 95% CI 1.10-3.07). Household food insecurity was also associated with women reporting COVID-related impacts to antenatal care; each one-unit increase in household food insecurity index (i.e., the number of household food insecurity indicators positively endorsed) was associated with an 18% increase in the odds of reporting COVID-19 to affect women's ability to access or attend ANC (aOR 1.18, 95% CI 1.06-1.32).

Table 5. Logistic regression adjusted odds ratios (95% confident intervals) of factors associated with women reporting COVID-19 to affect accessing or attending antenatal care (ANC) among women who delivered in 2020

	**Reported COVID-19 to affect accessing or attending ANC (N=540)**
**Age, years**	
Less than 25	Ref
25-29	0.57 (0.35-0.93)*
30-34	0.97 (0.56-1.69)
35 and older	0.82 (0.41-1.65)
**Married or partnered**	
No	Ref
Yes	0.92 (0.61-1.40)
**Educational attainment**	
Primary or less	Ref
Some secondary	2.36 (1.38-4.05)**
Secondary	3.23 (2.04-5.12)***
College/University	3.53 (1.82-6.84)***
**Currently employed**	
No	Ref
Yes	1.45 (0.87-2.42)
**Self-rated health status**	
Fair, poor, or very poor	Ref
Excellent, very good, or good	0.51 (0.34-0.75)**
**Parity**	
1	Ref
2 or more	1.84 (1.10-3.07)*
**Household food insecurity index**	1.18 (1.06-1.32)**

Note: ANC = antenatal care.
[1]Household food insecurity index denotes the number of household food insecurity indicators endorsed; possible scores range from 0 to 6.

For peer review only - http://bmjopen.bmj.com/site/about/guidelines.xhtml

*p<0.05, **p<0.01

Discussion

The primary objective of this study was to investigate the impacts of COVID-19 on ANC utilization comparing women who delivered before the pandemic to women who delivered during. Our findings suggest that COVID-19 was associated with delayed initiation of ANC after the first trimester and, consequently, inadequate ANC utilization. Compared to 20% among women who delivered before COVID-19 in 2019, only 14% of women who delivered during COVID-19 achieved adequate ANC utilization. Early initiation of ANC (i.e., initiation during the first trimester of gestation) is critical for timely detection and prevention of complications and receiving guidance on proper nutrition, immunization, treatment for infectious diseases, and the management of other chronic conditions (World Health Organization, 2016). Adequate utilization of ANC is also an important strategy to improve adverse birth outcomes, including preterm birth, low birth weight, and maternal and infant mortality (World Health Organization, 2016).

Interestingly, despite finding that women were more likely to delay ANC initiation during the pandemic, we found no difference in the total number of visits attended among women who delivered before COVID-19 to those who delivered during. It is possible that concern regarding potential risks of COVID-19 infection to them or their fetus motivated women to seek frequent care once care was initiated to properly monitor development. This may have occurred despite fears around contracting COVID-19 if going to health facilities or out into the community, as well as health facilities being closed or too busy as barriers to accessing or attending ANC. Furthermore, we do not know *where* women received antenatal care during COVID. It is possible that women who delivered during the pandemic were more likely to attend informal care networks than their counterparts who delivered before COVID-19 in instances where they were unable or unwilling to receive ANC within the formal healthcare system. Additional research is needed that explores women's decision-making regarding behaviors related to ANC utilization during the COVID-19 pandemic.

Nearly half of women who delivered during COVID-19 reported that the pandemic affected their ability to access or attend ANC. The most common reasons cited were related to facility factors, with over 80% combined reporting that COVID-19 affected their ANC use due to facilities being closed, too busy, or not accepting patients, fear of contracting the virus at the healthcare facility, and lack of trust in the healthcare facility. Other commonly reported barriers to ANC among our sample included fears related to contracting COVID-19 if going out into the community, an inability to pay for care, and difficulties related to lockdown measures. In Kenya, the pandemic may have resulted in significant health system breakdowns due to, in part, risk mitigation strategies (e.g., limiting in-person visits), limited supply of and cost for acquiring personal protective equipment, and healthcare worker strikes that forced facility closures. The expansion of telemedicine may be a helpful strategy for ensuring women achieve adequate utilization of ANC during pandemics and other public health emergencies by reducing barriers to care related to lockdowns, health system breakdowns, and psychosocial stressors (Osanan et al., 2020). One

For peer review only - http://bmjopen.bmj.com/site/about/guidelines.xhtml

quasi-experimental study conducted in Australia found that ANC service delivery via telemedicine during COVID-19 successfully reduced in-person visits by roughly 50% with no differences in the detection and management of common pregnancy complications (Palmer et al., 2021). Research is needed on the feasibility and of telemedicine in LMICs, particularly during public health emergencies, and interventions should focus on ensuring access to telemedicine visits are equitable by expanding access to those who attend public facilities and among families who are of lower socioeconomic status.

We also found that higher educational attainment, parity, and household food insecurity were positively associated with women's odds of reporting COVID-19 to have affected their ability to access or attend ANC. Previous research shows that women with higher educational attainment are more likely to attend ANC. Thus, our findings may reflect higher utilization among those with higher socioeconomic status, giving them more opportunities to encounter COVID-related barriers. It should be noted that a significantly lower prevalence of women who delivered during the pandemic were employed – this reflects the economic vulnerability that post-partum women face related to pregnancy and how the COVID-19 pandemic may exacerbate these existing inequities. Relatedly, our findings may also reflect differences in expectations of care across socioeconomic status that, in turn, influence perceived barriers to care (Connor et al., 2020). Previous studies find that women with higher educational attainment have higher expectations of maternity care than women with lower educational attainment (Galle et al., 2015). Furthermore, women with higher parity and higher household food insecurity may have been especially vulnerable to the economic implications of the pandemic, and thus, more likely to experience financial barriers to accessing or attending antenatal care. Even before the pandemic, parity and household food insecurity have been found to be significant predictors of inadequate antenatal care utilization (Gebremeskel et al., 2015; Magadi et al., 2000; Zeleke & Haymanot, 2020).

This study has some important limitations worth noting. First, the timing of ANC and number of ANC visits attended were self-reported, so recall bias may be present. Furthermore, our samples of women who delivered before and during COVID-19 may not be comparable despite sampling women who delivered during COVID-19 from the same catchment areas as those facilities where women who delivered before COVID-19 were sampled. Although we control for measured differences in individual and household level characteristics (e.g., differences in age, marital status, educational attainment) in regression analyses, it is possible that other unmeasured differences in sample characteristics explain study findings.

Conclusions

We find evidence that the pandemic may have resulted in an increased likelihood of delaying ANC after the first trimester, an important predictor of adverse pregnancy outcomes. Furthermore, half of women who delivered during COVID-19 reported that the pandemic affected their ability to access or attend antenatal care, with those with higher parity and household food insecurity having a higher odds of reporting barriers to care. Our findings point to several public health interventions that can minimize disruptions to healthcare utilization during pandemics and other public health, environmental, or political emergencies. First, the expansion of telemedicine for the delivery of antenatal care may be useful for reducing in-person

For peer review only - http://bmjopen.bmj.com/site/about/guidelines.xhtml

visits, particularly among those who are not deemed high-risk. Second, additional interventions, such as expanding access among low-income households to financial assistance, nutritional resources, and health insurance via the Kenyan National Hospital Insurance Fund (NHIF), may also have downstream effects on the receipt of adequate ANC. Lastly, community health workers may have a role to play in providing COVID-related information to pregnant and post-partum women in addition to providing maternal and child health-related services. Community health workers may also serve as an important conduit between women and their families and the healthcare system by referring them to appropriate care.

Ethics approval: Ethical clearance was received from the Kenya Medical Research Institute (KEMRI), Scientific and Ethics Review Unit (NON-KEMRI 702) and from the University of California Institutional Review Board (IRB #20-001421).

Funding statement: This work was supported by the Bill and Melinda Gates Foundation grant number INV-018586.

Competing interests: None to report.

Acknowledgments: We would like to thank the Innovation for Poverty Action field staff for data collection assistance, as well as Doris Njomo and Martina Gant for their support in reviewing survey measures and research questions.

Author contributions: AL contributed to data collection, led analysis and interpretation, and drafted the manuscript. JM contributed to data collection and writing of the manuscript. GG contributed to study design, data collection, interpretation, and writing of the manuscript. CM contributed to interpretation and writing of the manuscript. SK contributed to interpretation and writing of the manuscript. MS led study design and contributed to interpretation and writing of the manuscript.

Data sharing statement: Data will be shared upon reasonable request.

For peer review only

References

Burt, J., Ouma, J., Lubyayi, L., Amone, A., Aol, L., Sekikubo, M., Nakimuli, A., Nakabembe, E., Mboizi, R., Musoke, P., Kyohere, M., Namara, E., Khalil, A., & Doare, K. L. (2021). Indirect Effects of COVID-19 on Maternal, Neonatal, Child, Sexual and Reproductive Health Services in Kampala, Uganda. *MedRxiv*, 2021.04.23.21255940. https://doi.org/10.1101/2021.04.23.21255940

Chmielewska, B., Barratt, I., Townsend, R., Kalafat, E., Meulen, J. van der, Gurol-Urganci, I., O'Brien, P., Morris, E., Draycott, T., Thangaratinam, S., Doare, K. L., Ladhani, S., Dadelszen, P. von, Magee, L., & Khalil, A. (2021). Effects of the COVID-19 pandemic on maternal and perinatal outcomes: A systematic review and meta-analysis. *The Lancet Global Health*, *9*(6), e759–e772. https://doi.org/10.1016/S2214-109X(21)00079-6

Coates, J., Swindale, A., & Bilinsky, P. (2007). *Household Food Insecurity Access Scale (HFIAS) for Measurement of Food Access: Indicator Guide: Version 3* [Data set]. American Psychological Association. https://doi.org/10.1037/e576842013-001

Connor, J., Madhavan, S., Mokashi, M., Amanuel, H., Johnson, N. R., Pace, L. E., & Bartz, D. (2020). Health risks and outcomes that disproportionately affect women during the Covid-19 pandemic: A review. *Social Science & Medicine*, *266*, 113364. https://doi.org/10.1016/j.socscimed.2020.113364

Delamou, A., Ayadi, A. M. E., Sidibe, S., Delvaux, T., Camara, B. S., Sandouno, S. D., Beavogui, A. H., Rutherford, G. W., Okumura, J., Zhang, W.-H., & De Brouwere, V. (2017). Effect of Ebola virus disease on maternal and child health services in Guinea: A retrospective observational cohort study. *The Lancet. Global Health*, *5*(4), e448–e457. https://doi.org/10.1016/S2214-109X(17)30078-5

Galle, A., Van Parys, A.-S., Roelens, K., & Keygnaert, I. (2015). Expectations and satisfaction with antenatal care among pregnant women with a focus on vulnerable groups: A descriptive study in Ghent. *BMC Women's Health*, *15*(1), 112. https://doi.org/10.1186/s12905-015-0266-2

Gebremeskel, F., Dibaba, Y., & Admassu, B. (2015). Timing of First Antenatal Care Attendance and Associated Factors among Pregnant Women in Arba Minch Town and Arba Minch District, Gamo Gofa Zone, South Ethiopia. *Journal of Environmental and Public Health*, *2015*, e971506. https://doi.org/10.1155/2015/971506

Goyal, M., Singh, P., Singh, K., Shekhar, S., Agrawal, N., & Misra, S. (2021). The effect of the COVID-19 pandemic on maternal health due to delay in seeking health care: Experience from a tertiary center. *International Journal of Gynecology & Obstetrics*, *152*(2), 231–235. https://doi.org/10.1002/ijgo.13457

Islam, M. A., & Tabassum, T. (2021). Does antenatal and post-natal program reduce infant mortality? A meta-analytical review on 24 developing countries based on Demographic and Health Survey data. *Sexual & Reproductive Healthcare: Official Journal of the Swedish Association of Midwives*, *28*, 100616. https://doi.org/10.1016/j.srhc.2021.100616

Kassie, A., Wale, A., & Yismaw, W. (2021). Impact of Coronavirus Diseases-2019 (COVID-19) on Utilization and Outcome of Reproductive, Maternal, and Newborn Health Services at Governmental Health Facilities in South West Ethiopia, 2020: Comparative Cross-Sectional Study. *International Journal of Women's Health*, *13*, 479–488. https://doi.org/10.2147/IJWH.S309096

Kenya National Bureau of Statistics, Kenya Ministry of Health, National AIDS Control Council, Kenya Medical Research Institute, National Council for Population and Development, &

For peer review only - http://bmjopen.bmj.com/site/about/guidelines.xhtml

The DHS Program, ICF International. (2015). *Kenya Demographic and Health Survey 2014*. ICF International. https://dhsprogram.com/publications/publication-FR308-DHS-Final-Reports.cfm

Kuhnt, J., & Vollmer, S. (2017). Antenatal care services and its implications for vital and health outcomes of children: Evidence from 193 surveys in 69 low-income and middle-income countries. *BMJ Open*, *7*, e017122. https://doi.org/10.1136/bmjopen-2017-017122

Magadi, M. A., Madise, N. J., & Rodrigues, R. N. (2000). Frequency and timing of antenatal care in Kenya: Explaining the variations between women of different communities. *Social Science & Medicine (1982)*, *51*(4), 551–561. https://doi.org/10.1016/s0277-9536(99)00495-5

Osanan, G. C., Vidarte, M. F. E., & Ludmir, J. (2020). Do not forget our pregnant women during the COVID-19 pandemic. *Women & Health*, *60*(9), 959–962. https://doi.org/10.1080/03630242.2020.1789264

Palmer, K. R., Tanner, M., Davies-Tuck, M., Rindt, A., Papacostas, K., Giles, M. L., Brown, K., Diamandis, H., Fradkin, R., Stewart, A. E., Rolnik, D. L., Stripp, A., Wallace, E. M., Mol, B. W., & Hodges, R. J. (2021). Widespread implementation of a low-cost telehealth service in the delivery of antenatal care during the COVID-19 pandemic: An interrupted time-series analysis. *The Lancet*, *398*(10294), 41–52. https://doi.org/10.1016/S0140-6736(21)00668-1

Pervin, J., Moran, A., Rahman, M., Razzaque, A., Sibley, L., Streatfield, P. K., Reichenbach, L. J., Koblinsky, M., Hruschka, D., & Rahman, A. (2012). Association of antenatal care with facility delivery and perinatal survival – a population-based study in Bangladesh. *BMC Pregnancy and Childbirth*, *12*(1), 111. https://doi.org/10.1186/1471-2393-12-111

Ronen, K., McGrath, C. J., Langat, A. C., Kinuthia, J., Omolo, D., Singa, B., Katana, A. K., Ng'Ang'A, L. W., & John-Stewart, G. (2017). Gaps in Adolescent Engagement in Antenatal Care and Prevention of Mother-to-Child HIV Transmission Services in Kenya. *Journal of Acquired Immune Deficiency Syndromes (1999)*, *74*(1), 30–37. https://doi.org/10.1097/QAI.0000000000001176

Sudhinaraset, M., Landrian, A., Golub, G. M., Cotter, S. Y., & Afulani, P. A. (2021). Person-centered maternity care and postnatal health: Associations with maternal and newborn health outcomes. *AJOG Global Reports*, *1*(1), 100005. https://doi.org/10.1016/j.xagr.2021.100005

Tekelab, T., Chojenta, C., Smith, R., & Loxton, D. (2019). The impact of antenatal care on neonatal mortality in sub-Saharan Africa: A systematic review and meta-analysis. *PLOS ONE*, *14*(9), e0222566. https://doi.org/10.1371/journal.pone.0222566

Townsend, R., Chmielewska, B., Barratt, I., Kalafat, E., van der Meulen, J., Gurol-Urganci, I., O'Brien, P., Morris, E., Draycott, T., Thangaratinam, S., Doare, K. L., Ladhani, S., Dadelszen, P. von, Magee, L. A., & Khalil, A. (2021). Global changes in maternity care provision during the COVID-19 pandemic: A systematic review and meta-analysis. *EClinicalMedicine*. https://doi.org/10.1016/j.eclinm.2021.100947

Wairoto, K. G., Joseph, N. K., Macharia, P. M., & Okiro, E. A. (2020). Determinants of subnational disparities in antenatal care utilisation: A spatial analysis of demographic and health survey data in Kenya. *BMC Health Services Research*, *20*(1), 665. https://doi.org/10.1186/s12913-020-05531-9

For peer review only - http://bmjopen.bmj.com/site/about/guidelines.xhtml

World Health Organization. (2016). *WHO recommendations on antenatal care for a positive pregnancy experience*. World Health Organization. https://www.who.int/publications-detail-redirect/9789241549912

Yerger, P., Jalloh, M., Coltart, C. E. M., & King, C. (2020). Barriers to maternal health services during the Ebola outbreak in three West African countries: A literature review. *BMJ Global Health*, *5*(9), e002974. https://doi.org/10.1136/bmjgh-2020-002974

Zeleke, E. A., & Haymanot, A. N. (2020). Food Insecurity Associated with Attendance to Antenatal Care Among Pregnant Women: Findings from a Community-Based Cross-Sectional Study in Southern Ethiopia. *Journal of Multidisciplinary Healthcare*, *13*, 1415–1426. https://doi.org/10.2147/JMDH.S275601

For peer review only

For peer review only - http://bmjopen.bmj.com/site/about/guidelines.xhtml

Reporting checklist for cross sectional study.

Based on the STROBE cross sectional guidelines.

Instructions to authors

Complete this checklist by entering the page numbers from your manuscript where readers will find each of the items listed below.

Your article may not currently address all the items on the checklist. Please modify your text to include the missing information. If you are certain that an item does not apply, please write "n/a" and provide a short explanation.

Upload your completed checklist as an extra file when you submit to a journal.

In your methods section, say that you used the STROBE cross sectionalreporting guidelines, and cite them as:

von Elm E, Altman DG, Egger M, Pocock SJ, Gotzsche PC, Vandenbroucke JP. The Strengthening the Reporting of Observational Studies in Epidemiology (STROBE) Statement: guidelines for reporting observational studies.

		Reporting Item	Page Number
**Title and abstract**			
Title	#1a	Indicate the study's design with a commonly used term in the title or the abstract	1
Abstract	#1b	Provide in the abstract an informative and balanced summary of what was done and what was found	2
**Introduction**			
Background / rationale	#2	Explain the scientific background and rationale for the investigation being reported	4
Objectives	#3	State specific objectives, including any prespecified hypotheses	4
**Methods**			
Study design	#4	Present key elements of study design early in the paper	5
Setting	#5	Describe the setting, locations, and relevant dates, including periods of	5

For peer review only

		recruitment, exposure, follow-up, and data collection	
Eligibility criteria	#6a	Give the eligibility criteria, and the sources and methods of selection of participants.	5
	#7	Clearly define all outcomes, exposures, predictors, potential confounders, and effect modifiers. Give diagnostic criteria, if applicable	5
Data sources / measurement	#8	For each variable of interest give sources of data and details of methods of assessment (measurement). Describe comparability of assessment methods if there is more than one group. Give information separately for for exposed and unexposed groups if applicable.	5
Bias	#9	Describe any efforts to address potential sources of bias	6
Study size	#10	Explain how the study size was arrived at	6
Quantitative variables	#11	Explain how quantitative variables were handled in the analyses. If applicable, describe which groupings were chosen, and why	5-6
Statistical methods	#12a	Describe all statistical methods, including those used to control for confounding	6
Statistical methods	#12b	Describe any methods used to examine subgroups and interactions	n/a
Statistical methods	#12c	Explain how missing data were addressed	6
Statistical methods	#12d	If applicable, describe analytical methods taking account of sampling strategy	n/a
Statistical methods	#12e	Describe any sensitivity analyses	6
**Results**			
Participants	#13a	Report numbers of individuals at each stage of study—eg numbers potentially eligible, examined for eligibility, confirmed eligible, included in the study, completing follow-up, and analysed. Give information separately for for exposed and unexposed groups if applicable.	5
Participants	#13b	Give reasons for non-participation at each stage	n/a
Participants	#13c	Consider use of a flow diagram	n/a

For peer review only

Descriptive data	#14a	Give characteristics of study participants (eg demographic, clinical, social) and information on exposures and potential confounders. Give information separately for exposed and unexposed groups if applicable.	6-7
Descriptive data	#14b	Indicate number of participants with missing data for each variable of interest	n/a
Outcome data	#15	Report numbers of outcome events or summary measures. Give information separately for exposed and unexposed groups if applicable.	7-8
Main results	#16a	Give unadjusted estimates and, if applicable, confounder-adjusted estimates and their precision (eg, 95% confidence interval). Make clear which confounders were adjusted for and why they were included	8-9
Main results	#16b	Report category boundaries when continuous variables were categorized	n/a
Main results	#16c	If relevant, consider translating estimates of relative risk into absolute risk for a meaningful time period	n/a
Other analyses	#17	Report other analyses done—e.g., analyses of subgroups and interactions, and sensitivity analyses	8-9
**Discussion**			
Key results	#18	Summarise key results with reference to study objectives	11
Limitations	#19	Discuss limitations of the study, taking into account sources of potential bias or imprecision. Discuss both direction and magnitude of any potential bias.	12
Interpretation	#20	Give a cautious overall interpretation considering objectives, limitations, multiplicity of analyses, results from similar studies, and other relevant evidence.	12
Generalisability	#21	Discuss the generalisability (external validity) of the study results	12
**Other Information**			
Funding	#22	Give the source of funding and the role of the funders for the present study and, if applicable, for the original study on which the present article is based	13

The STROBE checklist is distributed under the terms of the Creative Commons Attribution License CC-BY. This checklist was completed on 13. December 2021 using https://www.goodreports.org/, a tool made by the EQUATOR Network in collaboration with Penelope.ai

For peer review only

BMJ Open

Effects of the COVID-19 pandemic on antenatal care utilization in Kenya: a cross-sectional study

Journal:	*BMJ Open*
Manuscript ID	bmjopen-2021-060185.R1
Article Type:	Original research
Date Submitted by the Author:	14-Feb-2022
Complete List of Authors:	Landrian, Amanda; University of California Los Angeles<br>Mboya, John; Innovations for Poverty Action Kenya<br>Golub, Ginger; Innovations for Poverty Action Kenya<br>Moucheraud, Corrina; University of California Los Angeles<br>Kepha, Stella; Kenya Medical Research Institute<br>Sudhinaraset, May; University of California Los Angeles
**Primary Subject Heading**:	Obstetrics and gynaecology
Secondary Subject Heading:	Public health, Obstetrics and gynaecology, Global health
Keywords:	COVID-19, OBSTETRICS, PUBLIC HEALTH

SCHOLARONE™
Manuscripts

I, the Submitting Author has the right to grant and does grant on behalf of all authors of the Work (as defined in the below author licence), an exclusive licence and/or a non-exclusive licence for contributions from authors who are: i) UK Crown employees; ii) where BMJ has agreed a CC-BY licence shall apply, and/or iii) in accordance with the terms applicable for US Federal Government officers or employees acting as part of their official duties; on a worldwide, perpetual, irrevocable, royalty-free basis to BMJ Publishing Group Ltd ("BMJ") its licensees and where the relevant Journal is co-owned by BMJ to the co-owners of the Journal, to publish the Work in this journal and any other BMJ products and to exploit all rights, as set out in our licence.

The Submitting Author accepts and understands that any supply made under these terms is made by BMJ to the Submitting Author unless you are acting as an employee on behalf of your employer or a postgraduate student of an affiliated institution which is paying any applicable article publishing charge ("APC") for Open Access articles. Where the Submitting Author wishes to make the Work available on an Open Access basis (and intends to pay the relevant APC), the terms of reuse of such Open Access shall be governed by a Creative Commons licence – details of these licences and which Creative Commons licence will apply to this Work are set out in our licence referred to above.

Other than as permitted in any relevant BMJ Author's Self Archiving Policies, I confirm this Work has not been accepted for publication elsewhere, is not being considered for publication elsewhere and does not duplicate material already published. I confirm all authors consent to publication of this Work and authorise the granting of this licence.

For peer review only

Effects of the COVID-19 pandemic on antenatal care utilization in Kenya: a cross-sectional study

Amanda Landrian[1], John Mboya[2], Ginger Golub[2], Corrina Moucheraud[1], Stella Kepha,[3] May Sudhinaraset[1]

[1]Jonathan and Karin Fielding School of Public Health, University of California, Los Angeles, Los Angeles, California, USA

[2]Innovations for Poverty Action, Nairobi, Kenya

[3]Kenya Medical Research Institute, Nairobi, Kenya

*Corresponding Author: Amanda Landrian, Jonathan and Karin Fielding School of Public Health, University of California, Los Angeles, 650 Charles E Young Drive South, Los Angeles, California, United States 90095; alandrian2@gmail.com

Word Count: 3,565

For peer review only

Abstract

Objective: The aim of this study was to assess the effects of COVID-19 on antenatal care (ANC) utilization in Kenya, including women's reports of COVID-related barriers to ANC and correlates at the individual and household levels.

Design: Cross-sectional study.

Setting: Six public and private health facilities and associated catchment areas in Nairobi and Kiambu Counties in Kenya.

Participants: Data were collected from 1,729 women, including 1,189 women who delivered in healthcare facilities before the COVID-19 pandemic (from September 2019-January 2020) and 540 women who delivered during the pandemic (from July through November 2020). Women who delivered during COVID-19 were sampled from the same catchment areas as the original sample of women who delivered before to compare ANC utilization.

Primary and secondary outcome measures: Timing of ANC initiation, number of ANC visits, and adequate ANC utilization were primary outcome measures. Among only women who delivered during COVID-19 only, we explored women's reports of the pandemic having affected their ability to access or attend ANC as a secondary outcome of interest.

Results: Women who delivered during COVID-19 had significantly higher odds of delayed ANC initiation (i.e., beginning ANC during the second vs. first trimester) than women who delivered before (aOR 1.72, 95% CI 1.24-2.37), although no significant differences were detected in the odds of attending 4-7 or ≥8 ANC visits versus <4 ANC visits, respectively (aOR 1.12, 95% CI 0.86-1.44 and aOR 1.46, 95% CI 0.74-2.86). Nearly half (n=255/540; 47%) of women who delivered during COVID-19 reported that the pandemic affected their ability to access ANC.

Conclusions: Strategies are needed to mitigate disruptions to ANC among pregnant women during pandemics and other public health, environmental, or political emergencies.

Strengths and limitations of this study

- This study provides evidence of COVID-related effects on antenatal care (ANC) utilization, a critical determinant of maternal and newborn health, among pregnant women in Kenya.
- This study leveraged existing survey data among post-partum women who delivered just prior to the declaration of the COVID-19 pandemic and recruited a new cohort of women who delivered during the pandemic to explore potential differences in ANC initiation, number of visits, and adequate ANC utilization between the two samples.
- Despite sampling women who delivered during the pandemic from the same catchment areas as those facilities where women who delivered before COVID-19 were sampled, the two samples may not be equivalent and, thus, unmeasured differences in sample characteristics may have contributed to the study findings.

For peer review only - http://bmjopen.bmj.com/site/about/guidelines.xhtml

- While we have assumed the COVID-19 pandemic to be the cause of changes to ANC utilization, the study design does not allow for formal assessment of causality.

Introduction

Timely and comprehensive antenatal care (ANC) is critical for the health of women and their newborns, allowing for the early detection and management of pre-existing conditions and pregnancy-related complications and reducing the risk of maternal and infant morbidity and mortality.[1–5] The World Health Organization recommends a minimum of eight ANC visits during a woman's pregnancy, with the first visit occurring during the first trimester of gestation;[5] however, significant barriers continue to exist for adequate ANC. In Kenya, site of the present study, only 58% of women reported attending at least 4 ANC visits in the most recent Demographic and Health Survey conducted in 2014.[6]

The COVID-19 pandemic has been extremely disruptive to health systems and services worldwide. Early data indicate that the pandemic has decreased women's use of ANC,[7] including in low- and middle-income countries (LMICs).[8–10] The COVID-19 pandemic has also worsened maternal and perinatal outcomes, particularly for vulnerable groups in LMICs,[11] but more information is needed about changes in care-seeking patterns during this period. Additionally, much of the available data have focused on overall volume of ANC services without differentiating between timing of initiation and total number of visits or examining heterogeneity in changes to better understand who was most affected by these pandemic-related disruptions. It is also important to understand the enduring effects of how COVID-19 may affect care-seeking. For example, even one year after the 2014-15 Ebola outbreak in West Africa, use of ANC had not yet returned to pre-outbreak levels.[12]

Previous research has highlighted the range of mechanisms through which a pandemic might affect health care-seeking behavior,[13] including individual-level factors such as reduced ability to pay for care if household income is affected by the pandemic, facility-level factors such as closures, health worker shortages, or entry requirements (use of masks and testing), and policy-level factors like restrictions on movement. Understanding how these complex factors may affect women's decisions around ANC is critical to developing appropriate interventions for encouraging care-seeking. Outside the context of a pandemic, Kenyan women from less-wealthy households, lower levels of educational attainment, and those of younger ages may be less likely to achieve adequate ANC.[14–16] It is important to understand how these social determinants of health, and other underlying risk factors, may intersect with the COVID-19 pandemic to affect antenatal care-seeking.

Using survey data among women who delivered before and during the COVID-19 pandemic, the primary objective of this paper is to assess the effects of COVID-19 on the utilization of ANC by examining whether there were reported changes in ANC use before versus during the pandemic. This paper also describes women's reports of the specific ways COVID-19 affected their ability

For peer review only - http://bmjopen.bmj.com/site/about/guidelines.xhtml

to attend ANC and the individual-, household-, and facility-level factors associated with women's likelihood of reporting COVID-19 to have impacted ANC access or utilization.

Methods

Study participants and recruitment

This study uses non-representative, cross-sectional data from two samples of participants: 1) Women recruited within seven days of delivery while admitted/upon discharge at one of six participating facilities (3 public hospitals, 2 private hospitals, and 1 health center) in Nairobi and Kiambu Counties from September 2019 through January 2020 (i.e., prior to the onset of the COVID-19 pandemic; n=1,197)[17] and 2) Women residing in catchment areas of these same six participating facilities, who delivered since pandemic-related restrictions were mandated in Kenya (i.e., from March 16, 2020; N=1,135).[18] The latter sample was recruited with the specific intent of understanding the effects of COVID-19 on maternal and newborn health by leveraging the existing data among the sample of post-partum women surveyed just prior to the start of the pandemic. Additional information about both samples, including eligibility and recruitment procedures, can be found in previous publications.[17,18] In short, eligible participants in both samples were those aged 15-49 years who had delivered a singleton birth within the specified timeframe and had access to a functional phone to allow for follow-up. Vaginal delivery was an additional eligibility criterion among the sample of women who delivered before COVID-19.[17] The sample of women who delivered before COVID-19 were conveniently sampled in partnership with facility staff working in the post-natal wards. All women in the post-natal ward during working hours who were still admitted or at discharge were approached to learn about the study and determine interest and eligibility; among the 1,357 women approached, a total of 1,197 consented and enrolled (88.2%) in this previous study which assessed women's receipt of person-centered maternity care and its association with maternal and newborn health outcomes. The sample of women who delivered during COVID-19 was conveniently sampled through engagement with community health volunteers and local village leaders and completed the survey in November 2020; among the 1,182 women contacted by phone, a total of 1,135 consented and enrolled in the study (96.0%).[18]

An experienced team of nine female enumerators participated in a three-day, virtual training on the study protocol and survey tools. This was followed by a one-day piloting exercise among 30 women for the enumerators to practice the study consent, assess and refine the survey flow, and test study logistics and quality check procedures. Participants were contacted by phone for both the consent and a one-time, 30-minute survey, though participants had the option for scheduling a separate time for the survey to be administered. For those unable to be reached, a total of 9 attempts were made across different days and times. Participants received the equivalent of approximately $1.00 (United States Dollar) of airtime as a token of appreciation.

Survey measures

The primary outcomes of interest were: timing of ANC initiation, total number of ANC visits, and adequate ANC utilization. Items on the number and timing of antenatal visits were adapted from the 2014 Kenya Demographic and Health Survey.[6] The timing of ANC initiation was measured by asking women approximately how many months or weeks pregnant they were when

For peer review only - http://bmjopen.bmj.com/site/about/guidelines.xhtml

they attended their first ANC appointment. A categorical variable was then created to capture if ANC began in the first, second, or third trimester. The total number of ANC visits was a categorical variable capturing whether women attended <4, 4-7, or ≥8 visits. Finally, information on the timing of ANC initiation and the total number of ANC visits was used to create a binary variable capturing whether women achieved adequate ANC utilization, defined as initiating ANC during the first trimester *and* attending at least 4 visits (1=yes, 0=no).

Among women who delivered during COVID-19 only, we explored whether women reported the pandemic to have affected their ability to access or attend ANC (1=yes, 0=no) as a secondary outcome of interest.

We also included information on individual and household sociodemographic characteristics, including age, marital status, educational attainment, employment status, self-rated health, and parity. Women who delivered during COVID-19 were asked about household food insecurity using the Household Food Insecurity Access Scale,[19] and assigned a score (ranging 0-6) reflecting how many household food insecurity indicators were endorsed (Cronbach's α=0.80). Women were also asked how the pandemic affected their ability to access or attend ANC.

Analyses

The analytic sample was first restricted to those with complete information on ANC measures (n=8/1,197 missing among women who delivered before COVID-19 and n=13/1,135 missing among women who delivered during COVID-19). To ensure that a substantial portion of the gestational period occurred during the pandemic (as opposed to a significant period of gestation occurring prior to the start of the COVID-19 pandemic and strictest lockdown measures) and would thus be vulnerable to potential COVID-related effects to ANC utilization, the sample of women who delivered during COVID-19 was further restricted to those who delivered from July 2020 through the end of the study period in November 2020. This resulted in an additional 582 women who delivered from March 16 through June 2020 being excluded and a final analytic sample of 1,189 women who delivered before and 540 women who delivered during COVID-19.

Data were analyzed using descriptive, bivariate, and multivariable statistics using StataSE version 15. Pearson chi-square tests were used to examine differences in the distribution of demographic characteristics and measures of ANC utilization across study samples. Multivariable logistic regression models were used to assess the relationship between study sample and timing of ANC initiation, number of ANC visits, and adequate ANC utilization, respectively, after controlling for individual level characteristics. Sensitivity analyses were conducted to examine the robustness of the models when restricting the sample of women who delivered during COVID-19 to those who delivered from August through November 2020 (N=372) and then September through November 2020 (N=234), respectively. These groups represent those whose gestational periods would have most significantly overlapped with the pandemic (i.e., most or all of their pregnancy occurred after March 16, 2020).

A multivariable logistic regression model was also used to assess factors associated with women reporting COVID-19 to affect accessing or attending ANC.

For peer review only - http://bmjopen.bmj.com/site/about/guidelines.xhtml

Ethical considerations

The Institutional Review Boards at the University of California, Los Angeles (UCLA) and Kenya Medical Research Institute (KEMRI) approved all study procedures and all women provided verbal consent.

Patient and public involvement

Patients and members of the public were not involved in the design of this research; however, members of the public, including community health volunteers and local village leaders in study catchment areas, were involved in the recruitment of women who had delivered during the pandemic. These members of the public were also provided a policy brief of key study findings to disseminate to stakeholders within their communities.

Results

Descriptive statistics of demographic characteristics stratified by study sample are shown in Table 1. Women who delivered during COVID-19 were older (33% vs. 21% aged at least 30 years; p<0.001), less likely to be married or partnered (69% vs. 83%; p<0.001), more likely to have a secondary education or higher (46% vs. 17%; p<0.001), and less likely to rate their health as excellent, very good, or good (67% vs. 87%; p<0.001) than women who delivered before COVID-19. A significantly lower proportion of women who delivered during the pandemic were employed at the time of the survey than those who delivered before (16% vs. 40%; p<0.001). Compared to women who delivered before COVID-19, those who delivered during were more likely to have 2 or more total births (74% vs. 63%; p<0.001). The mean household food insecurity index score for women who delivered during COVID was nearly 4 (standard deviation=2).

Table 1. Individual and household characteristics of women who delivered before and during the COVID-19 pandemic

Characteristic	**Women who delivered *before* COVID-19 N=1,189**	**Women who delivered *during* COVID-19 N=540**	**p-value[1]**
**Age (years)**			<0.001
Less than 25	576 (48.4)	197 (36.5)	
25-29	364 (30.6)	163 (30.2)	
30-34	170 (14.3)	124 (23.0)	
35 and older	79 (6.6)	56 (10.4)	
**Married or partnered (yes)**	983 (82.7)	374 (69.3)	<0.001
**Educational attainment**			<0.001
Primary or less	526 (44.2)	202 (37.4)	
Some secondary	467 (39.3)	91 (16.9)	
Secondary	165 (13.9)	189 (35.0)	
College/University	31 (2.6)	58 (10.7)	
**Currently employed (yes)**	476 (40.0)	88 (16.3)	<0.001
**Self-rated health status**			<0.001

For peer review only - http://bmjopen.bmj.com/site/about/guidelines.xhtml

Fair, poor, or very poor	157 (13.2)	179 (33.2)	
Excellent, very good, or good	1,032 (86.8)	361 (66.9)	
**Parity**			<0.001
1	441 (37.1)	141 (26.1)	
2 or more	748 (62.9)	339 (73.9)	
**Household food insecurity index[2], mean (SD)**	NA	3.7 (1.9)	NA

Note: Frequency (proportion) shown unless otherwise noted. Percentages may not add to 100 due to rounding.
SD = standard deviation. NA = Not applicable.
[1]Pearson chi-squared test
[2]Household food insecurity index denotes the number of household food insecurity indicators endorsed; possible scores range from 0 to 6.

Table 2 provides descriptive statistics of ANC utilization measures stratified by study sample. Most women in both study samples attended any ANC. A higher proportion of women who delivered before COVID-19 initiated ANC in the first trimester than women who delivered during (21% vs. 15%; p=0.002). No statistically significant differences in the number of ANC visits attended were detected across study samples; most women who delivered before and during COVID-19 attended 4 to 7 visits (61% vs. 60%, respectively). Finally, about 20% of women who delivered before the pandemic achieved adequate ANC utilization compared to 14% of women who delivered during (p=0.002).

Table 2. Utilization of antenatal care (ANC) among women who delivered before and during the COVID-19 pandemic

Characteristic	**Women who delivered *before* COVID-19 N=1,189**	**Women who delivered *during* COVID-19 N=540**	**p-value[1]**
**Attended any ANC, yes**	1,181 (99.3)	534 (98.9)	0.346
**Timing of ANC initiation**			0.002
First trimester	252 (21.2)	81 (15.0)	
Second trimester	777 (65.4)	425 (78.7)	
Third trimester or never	160 (13.5)	34 (6.3)	
**Number of ANC visits**			0.277
Less than 4	439 (36.9)	187 (34.6)	
4-7	717 (60.3)	331 (61.3)	
8 or more	33 (2.8)	22 (4.1)	
**Adequate ANC utilization[2], yes**	238 (20.0)	74 (13.7)	0.002

Note: Frequency (proportion) shown. Percentages may not add to 100 due to rounding.
ANC = antenatal care.
[1]Pearson chi-squared test
[2]Defined as initiating ANC during the first trimester *and* attending at least 4 ANC visits.

For peer review only - http://bmjopen.bmj.com/site/about/guidelines.xhtml

Results from logistic regression models assessing the relationship between study sample and measures of ANC utilization are shown in Table 3. After controlling for other individual level characteristics, women who delivered during the pandemic had significantly higher odds of initiating ANC in the second versus first trimester than women who delivered before (adjusted odds ratio [aOR] 1.72, 95% confidence interval [CI] 1.24-2.37). No significant differences in the odds of attending 4-7 or ≥8 ANC visits versus <4 ANC visits, respectively, were detected across the study samples. Women who delivered during COVID-19 had significantly lower odds of achieving adequate ANC utilization than women who delivered before after controlling for individual level characteristics (aOR 0.62, 95% CI 0.44-0.86). Findings did not substantively differ in sensitivity analyses restricting women who delivered during COVID-19 to those whose births occurred from August through November 2020 (N=372) and September through November 2020 (N=234), respectively (data not shown).

Table 3. Logistic regression adjusted odds ratios (95% confident intervals) of antenatal care (ANC) outcomes by study sample

	**Timing of ANC Initiation**		**Number of ANC Visits**		
Sample	**Second trimester vs. First trimester**	**Third trimester or never vs. First trimester**	**4-7 vs. Less than 4**	**8 or more vs. Less than 4**	**Adequate ANC Utilization**
Women who delivered *before* COVID-19	Ref	Ref	Ref	Ref	Ref
Women who delivered *during* COVID-19	1.72 (1.24-2.37)**	0.60 (0.36-1.00)	1.12 (0.86-1.44)	1.46 (0.74-2.86)	0.62 (0.44-0.86)**

Note: Timing of ANC initiation and number of ANC visits use multinomial logistic regression, while adequate ANC utilization uses multivariable logistic regression. All models are adjusted for individual characteristics including women's age, marital status, education, employment status, self-rated health status, and parity. ANC = antenatal care.
*p<0.05, **p<0.01

Women who delivered during COVID-19 were asked to report how the pandemic affected their ability to access or attend ANC (Table 4). Nearly half (47%) of all women reported *any* effects to ANC due to COVID-19. Among these women (N=255), the most reported effects included facilities being closed, too busy, or not accepting patients (61%), being scared to contract COVID-19 if going to a hospital or health facility (20%) or going out into the community (15%), an inability to afford care because of COVID-19 (15%), and COVID-related restrictions, such as curfews or mask mandates, hindering ANC access (12%).

Table 4. Reported COVID-related effects to antenatal care utilization among women who delivered during COVID

Effects	**Women who delivered *during* COVID-19 (N=540)**
**Reported COVID-19 to affect accessing or attending ANC**	
Yes	255 (47.2)

For peer review only - http://bmjopen.bmj.com/site/about/guidelines.xhtml

No	285 (52.8)
**Among those who reported COVID-19 to affect accessing or attending ANC (N=255)[1]**	
Facility was closed, too busy, or not accepting patients	156 (61.2)
Scared to get COVID if going to hospital/health facility	50 (19.6)
Could not afford care because of COVID	38 (14.9)
Scared to get COVID if going out into community	37 (14.5)
COVID-related restrictions (e.g., curfew, mask mandate)	30 (11.8)
Scared of police or other officials	8 (3.1)
Inability to pay for or find transportation	7 (2.8)
Do not trust health facility right now	4 (1.6)

Note: Frequency (proportion) shown. ANC = antenatal care.
[1]Responses are not mutually exclusive.

Table 5 provides results of the logistic regression model examining associations between individual and household level characteristics and the odds of women reporting COVID-19 to have affected their ability to access or attend ANC. A significant association was found between educational attainment and reporting COVID-related effects; increasing education was associated with increasing odds of reporting COVID-19 to affect women's ability to access or attend ANC compared to those with a primary education or less. Women who rated their health as excellent, very good, or good had an odds of reporting COVID-related effects to ANC that was about 50% lower than women who rated their health as fair, poor, or very poor (aOR 0.51, 95% CI 0.34-0.75). Compared to women with only one birth, women with 2 or more births had significantly higher odds of reporting COVID-19 to affect accessing or attending ANC (aOR 1.84, 95% CI 1.10-3.07). Household food insecurity was also associated with women reporting COVID-related effects to ANC; each one-unit increase in household food insecurity index (i.e., the number of household food insecurity indicators positively endorsed) was associated with an 18% increase in the odds of reporting COVID-19 to affect women's ability to access or attend ANC (aOR 1.18, 95% CI 1.06-1.32).

Table 5. Logistic regression adjusted odds ratios (95% confident intervals) of factors associated with women reporting COVID-19 to affect accessing or attending antenatal care (ANC) among women who delivered in 2020

	Reported COVID-19 to affect accessing or attending ANC (N=540)
**Age, years**	
Less than 25	Ref
25-29	0.57 (0.35-0.93)*
30-34	0.97 (0.56-1.69)
35 and older	0.82 (0.41-1.65)
**Married or partnered**	
No	Ref
Yes	0.92 (0.61-1.40)
**Educational attainment**	
Primary or less	Ref

For peer review only - http://bmjopen.bmj.com/site/about/guidelines.xhtml

Some secondary	2.36 (1.38-4.05)**
Secondary	3.23 (2.04-5.12)***
College/University	3.53 (1.82-6.84)***
**Currently employed**	
No	Ref
Yes	1.45 (0.87-2.42)
**Self-rated health status**	
Fair, poor, or very poor	Ref
Excellent, very good, or good	0.51 (0.34-0.75)**
**Parity**	
1	Ref
2 or more	1.84 (1.10-3.07)*
**Household food insecurity index**	1.18 (1.06-1.32)**

Note: ANC = antenatal care.
[1]Household food insecurity index denotes the number of household food insecurity indicators endorsed; possible scores range from 0 to 6.
*p<0.05, **p<0.01

Discussion

The primary objective of this study was to investigate the effects of COVID-19 on ANC utilization comparing women who delivered before the pandemic to women who delivered during. Our findings suggest that COVID-19 was associated with delayed initiation of ANC after the first trimester and, consequently, inadequate ANC utilization. Compared to 20% among women who delivered before COVID-19 in 2019, only 14% of women who delivered during COVID-19 achieved adequate ANC utilization. Furthermore, findings from sensitivity analyses, which used different cut-offs for overlap between the timing of ANC and COVID-19 and found no difference, suggest that COVID-19 was detrimental to the receipt of ANC even among women whose pregnancies may have only partially overlapped with the pandemic. Early initiation of ANC (i.e., initiation during the first trimester of gestation) is critical for timely detection and prevention of complications and receiving guidance on proper nutrition, immunization, treatment for infectious diseases, and the management of other chronic conditions.[5] Adequate utilization of ANC is also an important strategy to improve adverse birth outcomes, including preterm birth, low birth weight, and maternal and infant mortality.[5]

Interestingly, despite finding that women were more likely to delay ANC initiation during the pandemic, we found no difference in the total number of visits attended among women who delivered before COVID-19 to those who delivered during. It is possible that concern regarding potential risks of COVID-19 infection to them or their fetus motivated women to seek frequent care once care was initiated to properly monitor development. This may have occurred despite fears around contracting COVID-19, as well as health facilities being closed or too busy, as potential barriers to accessing or attending ANC. Furthermore, we do not know *where* women received ANC during COVID-19. It is possible that women who delivered during the pandemic were more likely to attend informal care networks than their counterparts who delivered before

For peer review only - http://bmjopen.bmj.com/site/about/guidelines.xhtml

COVID-19 in instances where they were unable or unwilling to receive ANC within the formal healthcare system. Additional research is needed that explores women's decision-making regarding behaviors related to ANC utilization during the COVID-19 pandemic.

Nearly half of women who delivered during COVID-19 reported that the pandemic affected their ability to access or attend ANC. The most common reasons cited were related to facility factors, with over 80% combined reporting that COVID-19 affected their ANC use due to facilities being closed, too busy, or not accepting patients, fear of contracting the virus at the healthcare facility, and lack of trust in the healthcare facility. Other commonly reported barriers to ANC among our sample included fears related to contracting COVID-19 if going out into the community, an inability to pay for care, and difficulties related to lockdown measures. There is strong evidence that COVID-19 has contributed to increases in stillbirths, miscarriages, maternal morbidity, and deaths.[11] Our data on reasons for how the pandemic affected women's ability to access or attend ANC may shed light on potential mechanisms for explaining increases in adverse maternal and neonatal health outcomes. In Kenya, the pandemic may have resulted in significant health system breakdowns due to, in part, risk mitigation strategies (e.g., limiting in-person visits), limited supply of and cost for acquiring personal protective equipment, and healthcare worker strikes that forced facility closures. The expansion of telemedicine may be a helpful strategy for ensuring women achieve adequate utilization of ANC during pandemics and other emergencies by reducing barriers to care related to lockdowns, health system breakdowns, and psychosocial stressors.[20] One quasi-experimental study conducted in Australia found that ANC service delivery via telemedicine during COVID-19 successfully reduced in-person visits by roughly 50% with no differences in the detection and management of common pregnancy complications.[21] Research is needed on the feasibility of telemedicine in LMICs, particularly during public health emergencies. Interventions should focus on ensuring access to telemedicine visits are equitable by expanding access to those who attend public facilities and among families who are of lower socioeconomic status.

Importantly, women with better self-rated health had significantly lower odds of reporting barriers to ANC than those with poorer self-rated health. Women with poorer health status may be more likely to avoid or delay care because of their increased vulnerability to COVID-19 infection and severe illness. However, because this group may also be more vulnerable to adverse pregnancy-related outcomes, early initiation of and routine ANC remains critical. During pandemics, it may be important to screen and identify pregnant women with poorer self-rated health to ensure continuity of ANC is maintained among this group.

We also found that higher educational attainment, parity, and household food insecurity were positively associated with women's odds of reporting COVID-19 to have affected their ability to access or attend ANC. Previous research shows that women with higher educational attainment are more likely to attend ANC. Thus, our findings may reflect higher utilization among those with higher socioeconomic status, giving them more opportunities to encounter COVID-related barriers. It should be noted that a significantly lower prevalence of women who delivered during the pandemic were employed – this reflects the economic vulnerability that post-partum women face related to pregnancy and how the COVID-19 pandemic may exacerbate these existing

For peer review only - http://bmjopen.bmj.com/site/about/guidelines.xhtml

inequities. Relatedly, our findings may also reflect differences in expectations of care across socioeconomic status that, in turn, influence perceived barriers to care.[22] Previous studies find that women with higher educational attainment have higher expectations of maternity care than women with lower educational attainment.[23] Furthermore, women with higher parity and higher household food insecurity may have been especially vulnerable to the economic implications of the pandemic, and thus, more likely to experience financial barriers to accessing ANC. Prior to the current pandemic, parity and household food insecurity were found to be significant predictors of inadequate ANC utilization, even in settings where ANC services at public facilities are available at no cost, as is the case in Kenya.[14,24,25] However, evidence suggests that women continue to incur out-of-pocket expenses during ANC visits throughout the country.[26] These unpredictable costs can render adequate ANC utilization unattainable for the most financially vulnerable, especially during public health emergencies.

This study has some important limitations worth noting. First, the timing of ANC and number of ANC visits attended were self-reported, so recall bias may be present. Furthermore, our samples of women who delivered before and during COVID-19 may not be completely comparable due to the place of recruitment (facility versus not), support in recruitment of sample (health facility providers versus community health volunteers) and timing of delivery (within seven days versus up to four months post-delivery). However, the sample is as similar as feasibly possible, including sampling women who delivered during COVID-19 from the same catchment areas as those facilities where women who delivered before COVID-19 were sampled. Although we control for measured differences in individual and household level characteristics (e.g., differences in age, marital status, educational attainment) in regression analyses, it is possible that other unmeasured differences in sample characteristics could have contributed to the study findings.

Conclusions

We find evidence that the pandemic may have resulted in an increased likelihood of delaying ANC after the first trimester, an important predictor of adverse pregnancy outcomes. Furthermore, half of women who delivered during COVID-19 reported that the pandemic affected their ability to access or attend ANC, with those with higher parity and household food insecurity and poorer self-rated health having a higher odds of reporting barriers to care. Our findings point to several public health interventions that can minimize disruptions to healthcare utilization during pandemics and other public health, environmental, or political emergencies. First, the expansion of telemedicine for the delivery of ANC may be useful for reducing in-person visits, particularly among those who are not deemed high-risk. Second, additional interventions, such as expanding access among low-income households to financial assistance, nutritional resources, and health insurance via the Kenyan National Hospital Insurance Fund (NHIF) may also have downstream effects on the receipt of adequate ANC. Lastly, community health workers may have a role to play in providing COVID-related information to pregnant and post-partum women in addition to providing maternal and child health-related services. Community health workers may also serve as an important conduit between women and their families and the healthcare system by referring them to appropriate care.

For peer review only - http://bmjopen.bmj.com/site/about/guidelines.xhtml

Contributors: AL contributed to data collection, led analysis and interpretation, and drafted the manuscript. JM contributed to data collection and writing of the manuscript. GG contributed to study design, data collection, interpretation, and writing of the manuscript. CM contributed to interpretation and writing of the manuscript. SK contributed to interpretation and writing of the manuscript. MS led study design and contributed to interpretation and writing of the manuscript.

Competing interests: None.

Funding: This work was supported by the Bill and Melinda Gates Foundation grant number INV-018586.

Acknowledgments: We would like to thank the Innovation for Poverty Action field staff for data collection assistance, as well as Doris Njomo and Martina Gant for their support in reviewing survey measures and research questions.

Ethics approval: Ethical clearance was received from the Kenya Medical Research Institute (KEMRI), Scientific and Ethics Review Unit (NON-KEMRI 702) and from the University of California Institutional Review Board (IRB #20-001421).

Data availability statement: Data will be shared upon reasonable request.

For peer review only

For peer review only - http://bmjopen.bmj.com/site/about/guidelines.xhtml

References

1. Islam MA, Tabassum T. Does antenatal and post-natal program reduce infant mortality? A meta-analytical review on 24 developing countries based on Demographic and Health Survey data. Sex Reprod Healthc. 2021 Jun;28:100616.

2. Kuhnt J, Vollmer S. Antenatal care services and its implications for vital and health outcomes of children: evidence from 193 surveys in 69 low-income and middle-income countries. BMJ Open. 2017 Nov 1;7:e017122.

3. Pervin J, Moran A, Rahman M, Razzaque A, Sibley L, Streatfield PK, et al. Association of antenatal care with facility delivery and perinatal survival – a population-based study in Bangladesh. BMC Pregnancy and Childbirth. 2012 Oct 16;12(1):111.

4. Tekelab T, Chojenta C, Smith R, Loxton D. The impact of antenatal care on neonatal mortality in sub-Saharan Africa: A systematic review and meta-analysis. PLOS ONE. 2019 Sep 13;14(9):e0222566.

5. World Health Organization. WHO recommendations on antenatal care for a positive pregnancy experience [Internet]. World Health Organization; 2016 [cited 2021 Aug 3]. Available from: https://www.who.int/publications-detail-redirect/9789241549912

6. Kenya National Bureau of Statistics, Kenya Ministry of Health, National AIDS Control Council, Kenya Medical Research Institute, National Council for Population and Development, The DHS Program, ICF International. Kenya Demographic and Health Survey 2014 [Internet]. Rockville, MD: ICF International; 2015 Dec [cited 2021 Nov 12]. Available from: https://dhsprogram.com/publications/publication-FR308-DHS-Final-Reports.cfm

7. Townsend R, Chmielewska B, Barratt I, Kalafat E, van der Meulen J, Gurol-Urganci I, et al. Global changes in maternity care provision during the COVID-19 pandemic: A

For peer review only - http://bmjopen.bmj.com/site/about/guidelines.xhtml

systematic review and meta-analysis. EClinicalMedicine [Internet]. 2021 Jun 19 [cited 2021 Aug 3]; Available from: https://www.scienceopen.com/document?vid=dba82c98-1f3a-4093-81f0-fc29d37ab1fd

8. Burt J, Ouma J, Lubyayi L, Amone A, Aol L, Sekikubo M, et al. Indirect Effects of COVID-19 on Maternal, Neonatal, Child, Sexual and Reproductive Health Services in Kampala, Uganda. medRxiv. 2021 Jun 25;2021.04.23.21255940.

9. Goyal M, Singh P, Singh K, Shekhar S, Agrawal N, Misra S. The effect of the COVID-19 pandemic on maternal health due to delay in seeking health care: Experience from a tertiary center. International Journal of Gynecology & Obstetrics. 2021;152(2):231–5.

10. Kassie A, Wale A, Yismaw W. Impact of Coronavirus Diseases-2019 (COVID-19) on Utilization and Outcome of Reproductive, Maternal, and Newborn Health Services at Governmental Health Facilities in South West Ethiopia, 2020: Comparative Cross-Sectional Study. Int J Womens Health. 2021;13:479–88.

11. Chmielewska B, Barratt I, Townsend R, Kalafat E, Meulen J van der, Gurol-Urganci I, et al. Effects of the COVID-19 pandemic on maternal and perinatal outcomes: a systematic review and meta-analysis. The Lancet Global Health. 2021 Jun 1;9(6):e759–72.

12. Delamou A, Ayadi AME, Sidibe S, Delvaux T, Camara BS, Sandouno SD, et al. Effect of Ebola virus disease on maternal and child health services in Guinea: a retrospective observational cohort study. Lancet Glob Health. 2017 Apr;5(4):e448–57.

13. Yerger P, Jalloh M, Coltart CEM, King C. Barriers to maternal health services during the Ebola outbreak in three West African countries: a literature review. BMJ Global Health. 2020 Sep 1;5(9):e002974.

For peer review only

For peer review only - http://bmjopen.bmj.com/site/about/guidelines.xhtml

14. Magadi MA, Madise NJ, Rodrigues RN. Frequency and timing of antenatal care in Kenya: explaining the variations between women of different communities. Soc Sci Med. 2000 Aug;51(4):551–61.

15. Ronen K, McGrath CJ, Langat AC, Kinuthia J, Omolo D, Singa B, et al. Gaps in Adolescent Engagement in Antenatal Care and Prevention of Mother-to-Child HIV Transmission Services in Kenya. J Acquir Immune Defic Syndr. 2017 Jan 1;74(1):30–7.

16. Wairoto KG, Joseph NK, Macharia PM, Okiro EA. Determinants of subnational disparities in antenatal care utilisation: a spatial analysis of demographic and health survey data in Kenya. BMC Health Services Research. 2020 Jul 18;20(1):665.

17. Sudhinaraset M, Landrian A, Golub GM, Cotter SY, Afulani PA. Person-centered maternity care and postnatal health: associations with maternal and newborn health outcomes. AJOG Global Reports. 2021 Feb 1;1(1):100005.

18. Sudhinaraset M, Landrian A, Mboya J, Golub G. The economic toll of COVID-19: A cohort study of prevalence and economic factors associated with postpartum depression in Kenya. Int J Gynaecol Obstet. 2022 Feb 12;

19. Coates J, Swindale A, Bilinsky P. Household Food Insecurity Access Scale (HFIAS) for Measurement of Food Access: Indicator Guide: Version 3 [Internet]. Washington, D.C.: American Psychological Association; 2007 [cited 2021 Aug 3]. Available from: http://doi.apa.org/get-pe-doi.cfm?doi=10.1037/e576842013-001

20. Osanan GC, Vidarte MFE, Ludmir J. Do not forget our pregnant women during the COVID-19 pandemic. Women & Health. 2020 Oct 20;60(9):959–62.

21. Palmer KR, Tanner M, Davies-Tuck M, Rindt A, Papacostas K, Giles ML, et al. Widespread implementation of a low-cost telehealth service in the delivery of antenatal

For peer review only

For peer review only - http://bmjopen.bmj.com/site/about/guidelines.xhtml

care during the COVID-19 pandemic: an interrupted time-series analysis. The Lancet. 2021 Jul 3;398(10294):41–52.

22. Connor J, Madhavan S, Mokashi M, Amanuel H, Johnson NR, Pace LE, et al. Health risks and outcomes that disproportionately affect women during the Covid-19 pandemic: A review. Social Science & Medicine. 2020 Dec 1;266:113364.

23. Galle A, Van Parys A-S, Roelens K, Keygnaert I. Expectations and satisfaction with antenatal care among pregnant women with a focus on vulnerable groups: a descriptive study in Ghent. BMC Women's Health. 2015 Dec 2;15(1):112.

24. Gebremeskel F, Dibaba Y, Admassu B. Timing of First Antenatal Care Attendance and Associated Factors among Pregnant Women in Arba Minch Town and Arba Minch District, Gamo Gofa Zone, South Ethiopia. Journal of Environmental and Public Health. 2015 Oct 12;2015:e971506.

25. Zeleke EA, Haymanot AN. Food Insecurity Associated with Attendance to Antenatal Care Among Pregnant Women: Findings from a Community-Based Cross-Sectional Study in Southern Ethiopia. J Multidiscip Healthc. 2020 Nov 2;13:1415–26.

26. Orangi S, Kairu A, Ondera J, Mbuthia B, Koduah A, Oyugi B, et al. Examining the implementation of the Linda Mama free maternity program in Kenya. The International Journal of Health Planning and Management. 2021;36(6):2277–96.

For peer review only

For peer review only - http://bmjopen.bmj.com/site/about/guidelines.xhtml

Reporting checklist for cross sectional study.

Based on the STROBE cross sectional guidelines.

Instructions to authors

Complete this checklist by entering the page numbers from your manuscript where readers will find each of the items listed below.

Your article may not currently address all the items on the checklist. Please modify your text to include the missing information. If you are certain that an item does not apply, please write "n/a" and provide a short explanation.

Upload your completed checklist as an extra file when you submit to a journal.

In your methods section, say that you used the STROBE cross sectionalreporting guidelines, and cite them as:

von Elm E, Altman DG, Egger M, Pocock SJ, Gotzsche PC, Vandenbroucke JP. The Strengthening the Reporting of Observational Studies in Epidemiology (STROBE) Statement: guidelines for reporting observational studies.

		Reporting Item	Page Number
**Title and abstract**			
Title	#1a	Indicate the study's design with a commonly used term in the title or the abstract	1
Abstract	#1b	Provide in the abstract an informative and balanced summary of what was done and what was found	2
**Introduction**			
Background / rationale	#2	Explain the scientific background and rationale for the investigation being reported	4
Objectives	#3	State specific objectives, including any prespecified hypotheses	4
**Methods**			
Study design	#4	Present key elements of study design early in the paper	5
Setting	#5	Describe the setting, locations, and relevant dates, including periods of	5

For peer review only

		recruitment, exposure, follow-up, and data collection	
Eligibility criteria	#6a	Give the eligibility criteria, and the sources and methods of selection of participants.	5
	#7	Clearly define all outcomes, exposures, predictors, potential confounders, and effect modifiers. Give diagnostic criteria, if applicable	5
Data sources / measurement	#8	For each variable of interest give sources of data and details of methods of assessment (measurement). Describe comparability of assessment methods if there is more than one group. Give information separately for for exposed and unexposed groups if applicable.	5
Bias	#9	Describe any efforts to address potential sources of bias	6
Study size	#10	Explain how the study size was arrived at	6
Quantitative variables	#11	Explain how quantitative variables were handled in the analyses. If applicable, describe which groupings were chosen, and why	5-6
Statistical methods	#12a	Describe all statistical methods, including those used to control for confounding	6
Statistical methods	#12b	Describe any methods used to examine subgroups and interactions	n/a
Statistical methods	#12c	Explain how missing data were addressed	6
Statistical methods	#12d	If applicable, describe analytical methods taking account of sampling strategy	n/a
Statistical methods	#12e	Describe any sensitivity analyses	6
**Results**			
Participants	#13a	Report numbers of individuals at each stage of study—eg numbers potentially eligible, examined for eligibility, confirmed eligible, included in the study, completing follow-up, and analysed. Give information separately for for exposed and unexposed groups if applicable.	5
Participants	#13b	Give reasons for non-participation at each stage	n/a
Participants	#13c	Consider use of a flow diagram	n/a

For peer review only

Descriptive data	#14a	Give characteristics of study participants (eg demographic, clinical, social) and information on exposures and potential confounders. Give information separately for exposed and unexposed groups if applicable.	6-7
Descriptive data	#14b	Indicate number of participants with missing data for each variable of interest	n/a
Outcome data	#15	Report numbers of outcome events or summary measures. Give information separately for exposed and unexposed groups if applicable.	7-8
Main results	#16a	Give unadjusted estimates and, if applicable, confounder-adjusted estimates and their precision (eg, 95% confidence interval). Make clear which confounders were adjusted for and why they were included	8-9
Main results	#16b	Report category boundaries when continuous variables were categorized	n/a
Main results	#16c	If relevant, consider translating estimates of relative risk into absolute risk for a meaningful time period	n/a
Other analyses	#17	Report other analyses done—e.g., analyses of subgroups and interactions, and sensitivity analyses	8-9
**Discussion**			
Key results	#18	Summarise key results with reference to study objectives	11
Limitations	#19	Discuss limitations of the study, taking into account sources of potential bias or imprecision. Discuss both direction and magnitude of any potential bias.	12
Interpretation	#20	Give a cautious overall interpretation considering objectives, limitations, multiplicity of analyses, results from similar studies, and other relevant evidence.	12
Generalisability	#21	Discuss the generalisability (external validity) of the study results	12
**Other Information**			
Funding	#22	Give the source of funding and the role of the funders for the present study and, if applicable, for the original study on which the present article is based	13

The STROBE checklist is distributed under the terms of the Creative Commons Attribution License CC-BY.
This checklist was completed on 13. December 2021 using https://www.goodreports.org/, a tool made by the EQUATOR Network in collaboration with Penelope.ai

For peer review only
